# Digital interaction in practice (DIP) between patient, general practitioner and home care services. Evaluation of a pilot study

**DOI:** 10.1080/02813432.2025.2597789

**Published:** 2025-12-10

**Authors:** Unn Sollid Manskow, Tor Magne Johnsen, Elin Breivik

**Affiliations:** ^a^ Norwegian Centre for eHealth Research, University Hospital of North Norway, Tromsø, Norway; ^b^ Faculty of Health Sciences, UiT The Arctic University of Norway, Tromsø, Norway

**Keywords:** General practitioner, home care services, patient, digital interaction, video conferences

## Abstract

**Objective:**

A shortage of healthcare personnel, an aging population and insufficient collaboration between services are highlighted as the greatest challenges in the Norwegian healthcare system. Digital Interaction in Practice (DIP) is a digital collaboration model involving the general practitioner (GP), home care nurses, patient and relatives. The aim is improved coordination and tailored treatment for frail elderly patients with complex needs. The model is developed in close collaboration with healthcare personnel and is being piloted in a large Norwegian municipality.

**Methods:**

This qualitative study included GPs, nurses, and managers in Trondheim municipality, who have actively participated in the development of the DIP model in cooperation with researchers. Focus groups were used to gather experiences of the development and early pilot phase from the views of GPs, nurses and managers. Data were analysed using Braun and Clarks thematic content analysis framework.

**Findings:**

DIP required changes in work practices and allocation of resources, particularly for home care nurses. Furthermore, the participants reported improved interdisciplinary collaboration and viewed DIP as a potential to increase the quality of healthcare services. The workflow chart describing the DIP model was useful but required adaption to local work practices. General practitioners and nurses valued closer collaboration and experienced a more holistic and patient-centred follow-up.

**Conclusion:**

The findings underline the importance of collaboration between GPs, nurses, managers and researchers in the development and local adaptation of the DIP model. Despite some resource challenges and a need for role clarification, DIP may impact resource use in the long term, improve collaboration and coordination among those involved, and achieve more comprehensive, individually tailored patient pathways. Further research on the DIP model′s impact is warranted following broader implementation.

## Introduction

In Norway, as in many other countries, the healthcare system faces major challenges such as a shortage of healthcare personnel, an aging population with complex conditions, poor collaboration between services, and unequal access to care [1,2]. To reduce pressure on the healthcare service, measures such as increased prevention, digitization, and new organizational models have been introduced to improve patient pathways for vulnerable groups [3–5]. These include better care for frail elderly and the use of team-based models such as the Patient-Centred Care Team and the Primary Health Care Team model [6,7]. Despite promising outcomes, implementing interprofessional collaboration is challenging, particularly involving general practitioners (GP), who already face an ever-increasing workload and more administrative tasks [3,8].

All Norwegian municipalities provide home care services to support individuals in living safely and independently in their own homes for as long as possible. The services include nursing and medical assistance, as well as practical and personal care. To ensure continuity, each user is typically assigned a primary contact person. In large municipalities, home care is organized in district-based units that deliver services to local residents.

Patients receiving home care constitute an important portion of GPs’ patient population, many of whom are frail elderly with complex conditions. Both GPs and home care nurses play key roles in assessing these patients, particularly when changes in health conditions occur. In the absence of effective collaboration tools, such assessments are often carried out independently [3], with communication primarily limited to e-messages. To address this gap, the Digital Interaction in Practice project (DIP) was initiated in general practice aiming to improve collaboration around vulnerable patient groups and enhancing the quality of care. The project involves the development of a new digital collaboration model, based on regular video meetings involving the GP, home care, patient, and relatives. Similar collaboration models have previously been shown to support more holistic and individualized care for frail elderly with complex needs [4,5,9].

In DIP, GPs, home care nurses and managers have been involved in developing the model to ensure it aligns with existing workflows and is integrated into the organization, which is an essential factor in the successful development and implementation of new digital services in the healthcare sector [10,11]. This has been an ongoing effort, with the model continuously evaluated and adjusted along the way.

The aim of DIP is to develop a useful and effective tool for GPs and home care services which improves collaboration, enhances the quality of healthcare and enables individually tailored treatment and follow-up of the frail elderly.

This article presents experiences with the development and early piloting of the DIP model in Trondheim municipality. The purpose is *to explore how DIP was experienced after the initial pilot phase in a selection of GPs and home care services in the municipality, as well as how managers responsible for organizing the healthcare service assessed the model.*

## Material and methods

### Development of the DIP model

The development of the DIP model followed a participatory design approach, in which key stakeholders (GPs, nurses and managers from Trondheim municipality) collaborated with researchers (USM, EB and TMJ) in a dynamic, iterative process to develop and pilot the model. Strong user involvement from stakeholders is a key driver in the DIP project, and important for research-driven knowledge production and innovation-driven service development [12]. Through regular collaboration meetings and workshops between researchers and the involved actors, the DIP model has been gradually developed and piloted in Trondheim municipality since mid-2022.

**Figure 1.**
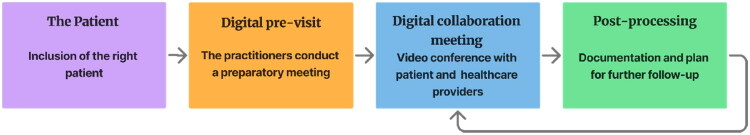
Flow chart of the four steps in the DIP-model.

Based on inputs from all actors, a flow chart outlining the model was created and continuously refined throughout the piloting phase (Figure 1). The flow chart serves as a dynamic working tool that provides a detailed description of the model’s four steps: (1) inclusion of patient and establishment of collaboration group, (2) digital pre-visit - preparatory meeting between the practitioners, (3) digital collaboration meeting between patient, practitioners, and relatives, (4) post-processing.

Home-dwelling complex and frail patients, usually with multimorbidity, with established follow-up from home care services were included in the piloting of DIP. All patients deemed eligible by the GPs and home care nurses received written and verbal information about DIP. Some patients declined participation in the video meetings; however, the exact number is currently unavailable. Agreements regarding patient inclusion and meeting scheduling were coordinated directly between GP and home care, primarily through e-messages. The supervising nurses, which are the nurses with the overall professional responsibility in each home care unit, were responsible for piloting DIP in their respective units. Either the supervising nurses or the patient’s primary contact organized the digital pre-visit and collaboration meetings, including distributing the meeting link. The digital meetings were conducted using Google Meet. During DIP meetings, the home care personnel participated from the patient’s home using portable data devices, while GPs participated from their offices. The model was solidly anchored in and supported by the municipal leadership and has been piloted since early 2023 in three of the municipality’s 12 home care units and with 10 GPs.

### Study design and data collection

The study was conducted by researchers with backgrounds as a GP (TMJ) a registered nurse (USM), and health science and health service research with experience in qualitative research methods (USM, EB).

USM and EB conducted three focus groups between June and August 2023. The most experienced users (GPs and nurses) of the DIP model were recruited as informants, several of whom also participated in the development of the model. All managers included in the study had participated in at least one workshop or meeting during the development phase. None of the informants dropped out or refused to participate after inclusion. The focus groups included four GPs, five nurses, and four managers from various levels in the municipality. All four GPs were specialists; three were female, and one was male. All nurses and managers were female. Each focus group consisted of participants from the same professional background, reflecting their different roles and responsibilities within the collaboration model. Focus groups were conducted to bring out experiences, reflection, and discussion from those involved [13] to inform further development of the model. All informants were aware that the researchers were participating in the development of the model. The focus groups including GPs and home care nurses were conducted in a meeting room at the city hall in Trondheim with USM and EB physically present, while the focus group with the municipal managers was conducted by USM and EB digitally via Microsoft Teams. No follow-up interviews were deemed necessary. A semi-structured interview guide [14] was used, covering the following main themes: Experience from participating in the development and use of DIP, perceived benefits, and challenges. The interviews lasted between 1 and 1.5 h and were audio-recorded and transcribed verbatim. Following the three focus group sessions, nearly all participants with experience in using the DIP model had been included.

## Analysis

The analysis followed the six steps of thematic reflexive analysis, an accessible method for exploring and interpreting qualitative interviews. The transcribed texts were read and coded separately by two authors (USM and EB) who subsequently participated collectively in the analyses of the data through thematic content analysis The transcribed data was first read thoroughly (USM and EB) to become familiar with the transcripts, build an initial understanding, then the text was ‘deconstructed’ and given new meaning through coding and generation of themes. In detail, the process involved the following six steps as described by Braun & Clark [15,16]: (1) Familiarizing ourselves with the dataset through reading and re-reading, while making notes and highlights in the transcript. (2) Coding the entire dataset using Nvivo 12. The codes were then transferred to tables in Word, where the next steps were performed. (3) Examining patterns in the codes and generating initial themes. (4) Checking the themes against the data to further develop and review the themes [5]. Developing a detailed analysis, defining, and focusing each theme, in addition to deciding on informative labels. A thematic categorization of the codes into eight themes was performed by USM and EB together: interdisciplinary collaboration – roles and collaboration, service quality, change of work practice, more holistic and patient-centred follow-up, organization and implementation, patients’ barriers, anchoring and scaling, and challenges. Finally, the themes were categorized into the four main findings: Development and introduction, Change of work practice and resource use, Interdisciplinary collaboration, and Service quality. We then moved on to step 6 weaving it all together and reporting the findings. Both USM and EB were involved in each step of the analysis process by having meetings and discussions before progressing to the next step. Although the analysis primarily followed a linear process, with each step building upon the previous one, there was also a need go back and forth between the steps. This aligns with the reflexive thematic analysis process, which typically involves recursion.

Our study was not guided by any specific pre-existing theory or conceptual framework; rather, the categories and main themes were derived inductively from the empirical data.

TMJ had the idea to the intervention and in his role as part-time GP, also piloted the model. To minimize potential bias, he was not involved in data collection or analysis but contributed to the introduction and the discussion sections.

### Ethical considerations

The study is approved by the Data Protection Officer (Internal review board), Centre for Research and Education at the University Hospital of North Norway (ref. no: 03035). According to the Data Protection Officer, the study falls out of scope according to the Norwegian Health Research Act and does not need an assessment or approval from the Regional Committee for Medical and Health Research Ethics. All participants were recruited based on voluntary participation and signed a written consent form prior to the interviews. The consent form explicitly stated the right to withdraw at any time without providing a reason. The data material was anonymized and handled securely according to the recommendations of the Data Protection Officer. Participant quotations presented in the results are only identified by profession to preserve anonymity.

## Results

Our analysis shows that the different actors experienced the development and piloting of the DIP model as a complex process caused by changes in work practices, adaption to a new workflow and allocation of resources, especially for the home care nurses. At the same time, improved interdisciplinary collaboration and the potential for increased quality in healthcare were highlighted. In the following sections, we present a more detailed description of the four main findings.

### Development and testing of the DIP-model were complex and demanding

The chart outlining the workflow in DIP was regarded as a useful tool by everyone involved. At the same time, both managers and home care nurses emphasized the importance of individual customization, as it has been crucial to adapt the model to the existing work practices within the different home care units.

*I think it will be important for implementation that the guidelines are not completely framed and very determined. That the [home care] units must be allowed to make their own adjustments to the model.* (Manager3)

All GPs and nurses found it positive that they had been involved in the development of DIP, and that the project was well anchored in the municipal leadership. The nurses emphasized the importance of feeling a sense of ownership of the collaboration model, and they had throughout the pilot phase taken responsibility for disseminating knowledge about DIP and implementing it in their home care units. As one nurse put it:

*It is important to have faith and desire to make it work, too. I think you depend on that in a project like this*. (Nurse3)

Nevertheless, it became clear that it has been challenging to reach out with information about DIP to other home care units and GPs, and the implementation has taken time.

*And we noticed this especially during the phase where we were supposed to disseminate the project to GPs that didn’t work with the [home care] units that originally were part of the project. There was some uncertainty and some GPs who ended up calling a unit that hadn’t heard of the project*. (Manager2)

### Change of work practice and resource use – time-consuming but effective

GPs pointed out that DIP entailed some changes in their work practices. The video meetings were perceived as resource intensive as they lasted longer than a standard consultation, but at the same time, they clarified the patient’s situation and a plan for further follow-up were made. Several GPs believed that this could contribute to reducing the number of future e-messages and other inquiries, and possibly save both time and resources:

*And to achieve the same result, it would have taken many e-messages, and perhaps frustrated and worried relatives, and yes… So, a very elegant way to solve it.* (GP1)

DIP can save time and resources for home care units because they can carry out many of the necessary measurements, for instance blood pressure or blood tests, in the patient’s home and, through collaboration with the GP, make a plan for further follow-up of the patient.

*We can carry out many of their tests at home. If you add up all these home visits and e-messages and the time spent, it kind of results in savings that we can handle everything in one meeting.* (Manager3)

One of the main challenges, however, was that several supervising nurses spent a lot of time and resources on DIP. It was a break with their normal workflow to conduct the meeting in the patient’s home, as the specialist nurse normally doesn’t perform home visits. As a result, several units began involving the patients’ primary contacts in DIP meetings. In these units, it was at times some uncertainty about who is responsible for conducting DIP.

The informants emphasized that to ensure that DIP works smoothly in everyday practice, more experience with the collaboration meetings is needed. Additionally, the various units must provide training to a greater number of the patients’ primary contacts.

*There is a bit of “learning by doing” as well, so I feel that the more included patients and the longer you keep at it, the better you get.* (Nurse2)

### Interdisciplinary collaboration led to better mutual understanding

Especially nurses emphasized that the collaboration with GPs increased both their professional competence and their understanding of the GPs’ work situation. The GPs expressed the same and emphasized that they had gained a broader and more comprehensive view of the municipal health and care service than they had previously. In addition, they reported increased appreciation of the extensive work carried out by home care for the patients.

*But primary healthcare, it’s not just me alone, I’m just a small piece in the big picture, right? […] and so in DIP, it’s the first time in a long time that I feel like a part of something larger. (GP1)*

Both GPs and nurses experienced that DIP improved their collaboration, which they highly valued. Both groups emphasized that they are now more than before “speaking with one voice” and that the digital pre-visit, helped establish a shared understanding of the patient’s situation and care plan. The different roles were perceived as clear. Additionally, home care nurses noted that they now “borrow” some of the GPs’ authority in patient encounters.

*We see the impact of having the GP present there [in DIP meetings] and can support measures that we think are good. That’s very positive.* (Nurse3)

### Potential for better quality of health and care services?

Across the focus groups, improved service quality was highlighted as a major potential of DIP. Informants experienced that the collaboration model supports a more holistic and patient-centred follow-up, among other things, because GPs and home care nurses, through a common understanding of the patient’s challenges, get ahead of the patient’s issues. They also considered that patients who would otherwise struggle to attend in-person consultations can benefit greatly from seeing their GP in a video conference.

*And it’s really nice because it turns into an open conversation that is not about one single issue. I think thatit isimportant for the patient. So, I’ve had meetings with doctors where they’ve said: “Oh, is that your problem? I wasn’t aware of that.”* (Nurse2)

The nurses were familiar with videoconferencing in general, as well as the specific video conferencing tool used. However, some noted that video conferences could be perceived as unfamiliar and difficult for some patients. Cognitive impairment, reduced vision, and hearing loss are factors that could make it difficult to follow the conversation. Due to limited digital competence among some of the patients, home care personnel always brought a laptop and mobile internet connection to the patient’s home and participated in the video conferences with the patient.

The preventive aspect of DIP was seen as having a potential to enhance patients’ quality of life. Furthermore, the possibility of preventing admissions to institutions such as hospitals or nursing homes was highlighted as a very positive outcome.

*I think we can prevent quite a few admissions, and [there are] vulnerable patients that we can keep at home for longer. That’s also what the municipality wants, and what we are working toward, that they should be able to stay at home for as long as possible. I think that if we have frequent meetings like this, we can achieve that*. (Nurse3).

## Discussion

This article presents the experiences of GPs, nurses, and managers in Trondheim municipality who have been actively involved in the development and early piloting of the DIP digital collaboration model. The findings indicate predominantly positive experiences with DIP, alongside anticipated future benefits such as enhanced interdisciplinary collaboration, improved quality of patient follow-up, more efficient use of resources, and contributions to disease prevention. At the same time, the findings highlight the complexity of implementing new collaboration models in healthcare and that processes related to both change in work practice and dissemination require significant effort from all involved actors.

In line with studies related to the acceptance and scaling of digital interventions, active participation and collaboration between all the involved parties and researchers have been central in all phases of the project [12,17]. The dynamic and detailed flow chart that describes procedures and division of responsibilities, was considered useful because it was adapted and adjusted based on the experiences of GPs and home care nurses with DIP. Socio-technical conditions, that is, the interaction between technology, people, and organization, are often more important for successful implementation than the technology itself [18]. Our findings show that the informants value the opportunity for adaptations of the model based on local organization and workflow, as well as the use of technology.

GPs and home care nurses reported that DIP required increased resource use in the short term; however, the video conferences provided better clarification of the patient’s condition compared to traditional consultations or e-messages. Consequently, DIP may hold potential for reducing resource use over time, for example by decreasing the number of e-messages and contact points. At this early stage of the intervention, resource use has not yet been examined, representing a topic for further research. As highlighted in previous studies, effective collaboration in video conferences can be challenging, often due to unclear division of responsibilities [19]. To address this, we established detailed descriptions of roles and responsibilities in the DIP model, including conducting and documenting meetings. Informants also emphasized the importance of the pre-visit, digital meetings between involved health care personnel to discuss and prepare prior to engaging the patient. Nonetheless, the division of responsibilities in some home care units remained unclear, which shows that introducing new services is often more complex and resource-intensive than expected [11,20].

DIP has established a new collaborative arena for the follow-up of frail patients, enabling GPs and home care nurses to collaborate in a new way. Our findings indicate that video conferences can support ongoing collaboration by facilitating information exchange between healthcare personnel across organizational boundaries [21]. Home care nurses viewed the digital meetings as an opportunity to enhance their professional competence through closer interactions with GPs, while GPs emphasized the potential for improved collaboration in managing patients with both complex medical needs and time-intensive follow-up. They also reported gaining greater insight into the working methods and organizational structures of other primary healthcare services in the municipality. In line with previous research, shared insight between professionals improves communication, assessments, and enables more targeted treatment planning [22]. A recent literature review highlights that good communication tools and recognition of others’ skills foster collaboration, whereas inadequate communication, time constraints, and insufficient training are barriers [23]. These findings align with the experiences of GPs, home care nurses, and municipal managers during the pilot phase of DIP.

Healthcare services in Norway are changing from a diagnosis-specific to a person-centred focus [6,24], aiming to provide patients with more holistic and coordinated health care services. Further research on both experiences with and effects of DIP will be important to follow the processes related to its dissemination and further development. As of 2025, the piloting of DIP has been scaled up to include all home care units and GPs in the municipality, with the model being continuously developed by its users. Future studies assessing the impact of DIP following large-scale implementation is warranted for the next years to come. Based on the findings from the early pilot phase, DIP has the potential to improve healthcare quality by facilitating more person-centred and individually tailored services. The model enables GPs and home care nurses to develop a shared understanding of the patient’s condition, preferences, and needs, thereby supporting more coordinated decision-making and follow-up.

### Study strengths and weaknesses

A key strength of this study lies in its focus on developing an innovative model for interaction between the GP, home care nurse, and patient, addressing the needs for enhanced collaboration, prevention, resource efficiency, and digitization in the care of an aging population with complex conditions, as outlined in the Norwegian Government’s white papers (1). The involved health personnel were actively engaged throughout the process, working closely with the researchers in regular meetings. The development of the model would not have been possible without the active involvement and contextual knowledge of the participating healthcare professionals and managers. The use of a qualitative design with focus groups was chosen to explore in depth the initial experiences and perspectives on the DIP model in clinical practice.

Another strength is the composition of the interdisciplinary research team, comprising a GP (TMJ), a nurse (USM), and all authors’ extensive experience in health services research and the implementation of digital interventions in municipal healthcare services. TMJ’s dual role as a GP and researcher brought valuable insight from long-standing clinical practice in Trondheim and firsthand experience with the challenges of insufficient interaction between different actors involved in the care of frail elderly patients. A potential limitation of the study is that the focus groups and subsequent data analysis were conducted by USM and EJ, who were both actively involved in the development of DIP.

A potential limitation of this study is that the focus groups were conducted separately for each professional group rather than in mixed settings. This approach may have constrained opportunities for interprofessional dialogue and the exchange of perspectives across disciplines. However, at this early stage of evaluating the DIP model, it was considered important to capture the distinct viewpoints of each profession to identify areas for improvement in the further development and implementation of the model. The relatively small number of participants may also be a limitation; nevertheless, our primary aim was to capture early experiences, and our informants represented nearly all health personnel with experience using DIP. Furthermore, several participants had a strong sense of ownership of the project and regular interaction with the researchers, which may have introduced some bias. Conversely, the active involvement of such key stakeholders was essential for anchoring the model within the municipality and promoting its dissemination.

Another limitation is that, in this early pilot phase, patients and relatives were not included as focus group informants. Although several patients and some relatives had used the model, the home care nurses did not succeed in recruiting patients or relatives as informants at this point The exploration of patients’ and relatives’ experiences will be a central focus in the next phase of the project, once the DIP model has been more fully implemented within the municipality.

The findings from this study may have applicability beyond the immediate context, particularly for the early-stage evaluation of digital interventions designed to enhance interprofessional interaction, whether within hospital settings or across the interface between primary and specialized healthcare services.

## Conclusion

This article presents early experiences with the DIP digital collaboration model. DIP facilitates a shared understanding between home care nurses and GPs when assessing the health status of frail elderly patients. Aligned with the government’s goal to reduce pressure on healthcare services, through prevention, digitization, and new forms of collaboration in primary care, DIP holds potential to support more holistic patient pathways for vulnerable groups. Further research is planned to explore the scaling of the model and its benefits for patients.

## Supplementary Material

Supplementary file nterview guides.docx
