# Supplementary material for: Digital interaction in practice (DIP) between patient, general practitioner and home care services. Evaluation of a pilot study
Source: Scand J Prim Health Care. 2025 Dec 10;44(1):1–9. doi: 10.1080/02813432.2025.2597789 (PMC12918335; doi:10.1080/02813432.2025.2597789)
Supplement: Supplementary file nterview guides.docx [file IPRI_A_2597789_SM0980.docx]

**Interview guides**

**Managers**

1. Current challenges in the municipal healthcare services, and how can DIP help address these challenges?

- The alignment of DIP with the strategy for health and care services

1. The anchoring of DIP within the municipality and home care units
2. Experiences with being involved in shaping and/or facilitating DIP
3. Resources spent to develop and test DIP
4. Potential benefits of DIP
5. Going forward: further dissemination in the municipality

**Nurses**

1. Current challenges in the municipal healthcare services regarding frail and complex patients, and how can DIP help address these challenges?
2. Experience with DIP meetings (pre-visits and DIP meetings the patient present) and the DIP flowchart
3. Experience with collaboration with GPs in DIP
4. Resources needed to carry out DIP meetings
5. Perceived benefits of DIP
6. Challenges with DIP
7. The technical solution for planning and execution of video conferences

**GPs**

1. Current challenges in the municipal healthcare services regarding frail and complex patients, and how can DIP help address these challenges?
2. Experience with DIP meetings (pre-visits and DIP meetings the patient present) and the DIP flowchart
3. Experience with collaboration with home care nurses in DIP
4. Resources needed to carry out DIP meetings
5. Perceived benefits of DIP
6. Challenges with DIP
7. The technical solution for video conferences
